# Synthesis, Molecular Docking and Biological Evaluation of A-Ring-Carborane-Vitamin D Analogues

**DOI:** 10.3390/molecules30122637

**Published:** 2025-06-18

**Authors:** Rocío Otero, Samuel Seoane, Xoán Fernández-Domínguez, Maxime Bourguet, Sarah Cianférani, Carole Peluso-Iltis, Miguel A. Maestro, Román Pérez-Fernández, Natacha Rochel, Antonio Mouriño

**Affiliations:** 1 Ignacio Ribas Research Laboratory, Department of Organic Chemistry, University of Santiago de Compostela, 15782 Santiago de Compostela, Spainxoan.fernandez.dominguez@rai.usc.es (X.F.-D.); antonio.mourino@usc.es (A.M.); 2 Department of Physiology-Center for Research in Molecular Medicine and Chronic Diseases (CIMUS), University of Santiago de Compostela, 15707 Santiago de Compostela, Spain; 3 Laboratoire de Spectrométrie de Masse BioOrganique, Université de Strasbourg, CNRS, IPHC UMR 7178, 67037 Strasbourg, France; 4 Infrastructure Nationale de Protéomique ProFI-UAR2048, 67087 Strasbourg, France; 5 Institut de Génétique et de Biologie Moléculaire et Cellulaire (IGBMC), Université de Strasbourg, CNRS, Inserm, UMR 7104-UMR-S 1258, 67400 Strasbourg, France; 6 Departamento de Química-CICA, Universidad de A Coruña, 15071 A Coruña, Spain

**Keywords:** vitamin D_3_ analogues, vitamin D_3_ receptor, A-ring-carboranes, synthesis, docking, biological activity

## Abstract

The active form of vitamin D_3_, 1α,25-dihydroxyvitamin D_3_ (1,25D_3_), regulates a number of physiological and pathological processes, including cell proliferation and differentiation. Thousands of analogues of 1,25D_3_ have been developed with the aim of selective effects for medical use. Here we describe the synthesis of two new unconventional vitamin D analogues bearing A-ring modifications with *ortho*-carborane (dicarba-*o*-*closo*-1,2-dodecaborane) units. The ligands function as agonists for VDR with similar antiproliferative activities as 1,25D_3_. Whereas mice treated with the analogues **4** and **5** exhibited similar hypercalcemic activities as 1,25D_3_, only compound **4** and 1,25D_3_ induced the strong activation of CYP24A1 mRNA expression but not compound **5**.

## 1. Introduction

**Figure 1:**
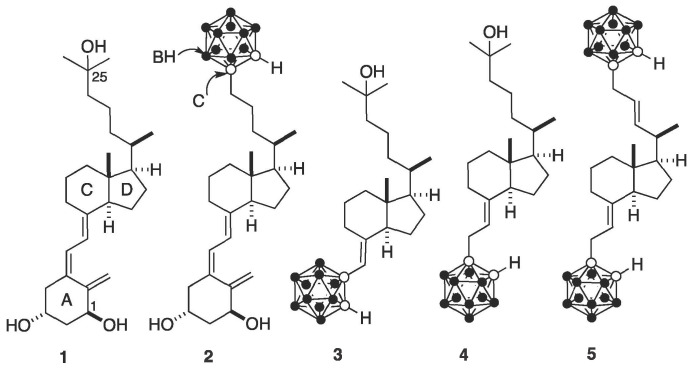
1α,25-dihydroxyvitamin D_3_ (**1**), known carborane-vitamin D_3_ analogues **2** and **3**, and target carborane-vitamin D_3_ analogues **4** and **5**.

Vitamin D_3_, before eliciting its physiological functions, undergoes two hydroxylations, first in the liver and then in the kidney, to produce 1α,25-dihydroxyvitamin D_3_ (1,25D_3_, Figure 1), which is the biologically active vitamin D_3_ form. This secosteroidal hormone, through binding to the vitamin D receptor (VDR), regulates a number of physiological processes including calcium homeostasis, cell proliferation and differentiation, and immunomodulation [1,2,3]. Recent research has been devoted to the development of 1,25D_3_ analogues with selective effects for medical use and some of them have already found clinical application [4,5,6,7]. We previously synthesized compound **2** (Figure 1) as the first secosteroidal analogue of vitamin D_3_, which contains an *o*-carborane unit at the side chain [8]. In comparison with the natural hormone 1,25D_3_, the analogue **2**, which lacks the side-chain-C25-OH group, binds strongly to the VDR and induces similar biological activities though with reduced toxic calcemic effects. The carborane unit mimics the interaction of the side-chain-C25-OH of 1,25D_3_ with the VDR and presents additional hydrophobic contacts between the boron atoms and important residues of the H12 helix. Aside from compound **2**, Kittaka and coworkers have reported the synthesis of carboranyl-vitamin D_3_ analogue **3**, that contains a carboranyl unit in the bottom part of the vitamin D_3_ skeleton [9]. In addition, a series of carborane-based non-secosteroidal VDR ligands have been shown to exhibit comparable activity than secosteroidal compounds [10].

The interesting biological properties of carboranyl vitamin D_3_ analogue **2** led us to undertake a programme aimed at the design, synthesis, and evaluation of new vitamin D_3_ analogues bearing carboranyl units at different positions of the vitamin D_3_ skeleton. Here we report on the design, synthesis, and evaluation of vitamin D analogues **4** and **5** (Figure 1).

## 2. Results and Discussion

### 2.1. Docking

**Figure 2:**
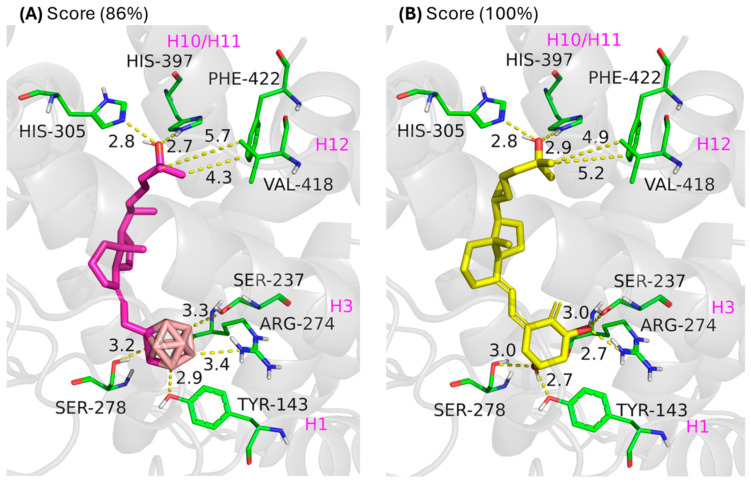
Docking on target vitamin D analogue **4**. (**A**) Docked structure of analogue **4** (magenta, left) bound to hVDR LBD (PDB code: 1DB1). The side chains of amino acid residues of the LBP are shown in green with the sequence code. The main interactions of analogue **4** with the amino acid residues of hVDR LBD are indicated by dotted lines and the distances are shown in Angstroms (Å). Helices are labelled in pink. Relative Scoring (%) in comparison with that of 1,25D_3_ (**1**) (100%) is shown in parenthesis. (**B**) Docked structure of the natural hormone 1,25D_3_ (yellow, right). The superimposition of analogue **4** with 1,25D_3_ (**1**) is shown in Figure SI-1.

Molecular docking studies were performed using Gaussian View 09 [11] and GOLD [12] software on vitamin D_3_ analogue **4**, targeting the crystal structure of the *h*VDR ligand-binding domain (LBD) (PDB ID: 1DB1) [13], which lacks the flexible insertion domain in the LBD and has been successfully crystallized in complex with several vitamin D_3_ analogues of 1,25D_3_ (**1**). The GOLD software assigns a score to the analogue based on its interactions with various amino acid residues within the active site pocket, accounting for steric hindrance, torsional angles, and other structural parameters. The more favourable these variables are, the more stable the resulting ligand–receptor complex. Once the scores are obtained, they are normalized using the natural hormone 1,25D_3_ as the reference (100%). To validate the results, a theoretical model of the hormone was included as an external reference. Analogue **4** achieved a docking score of 86% relative to 1,25D_3_. Ligand **4** adopts the typical elongated conformation of 1,25D_3_ in the binding pocket (Figure 2A) [13,14]. Key hydrogen bonds were observed between the C25-hydroxyl group of the side chain and residues His305 and His397, analogous to those seen with 1,25D_3_ (Figure 2B). Other significant hydrogen bonding interactions typically formed between the A-ring of 1,25D_3_ and residues Tyr143, Ser237, Arg274, and Ser278 were instead replaced by interactions between these same residues and the hydrogen atoms bound to the boron atoms of the carborane unit substituting the A-ring. Molecular docking calculations on analogue **5** using Gaussian View 09 and GOLD software failed presumably due to steric hindrance imparted by the two *o*-carborane residues.

### 2.2. Retrosynthetic Analysis of Target Analogues and **5**

**Scheme 1:**
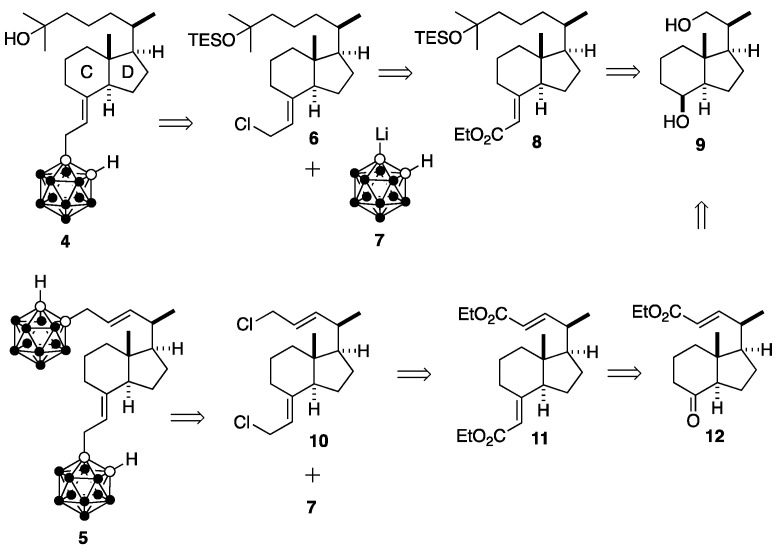
Retrosynthesis of target vitamin D_3_ analogues **4** and **5**.

The idea of introducing the carboranic units replacing the A-ring and the side chain of 1,25D_3_ aimed to mimic the interactions of the hydroxyls of the natural hormone with the amino acids of the receptor. The installation of the *ortho*-carboranyl unit of the target analogue **4** would be achieved by reaction of carboranyllithium **7** with allyl chloride **6**, which would arise from protected α,β-unsaturated ester **8**, which in turn would be prepared in 60% yield over 4 steps from Inhoffen-Lythgoe diol (**9**) (Scheme 1). The installation of both *ortho*-carboranyl units in the target analogue **5** would be achieved by reaction of carboranyllithium **7** with dichloride **10**, which would arise conventionally in two steps from the α,β-unsaturated diester **11**, which would be prepared by the Wadsworth–Horner–Emmons reaction on the ketone **12**. This ketone would be prepared in several conventional steps from Inhoffen-Lythgoe diol (**9**), which contains five stereocenters and the typical C and D rings of the hormone 1α,25-dihydroxyvitamin D_3_ (**1**, Figure 1) and most of its analogues. Diol **9** can be prepared by total synthesis [15,16,17] or more conveniently by reductive-ozonolysis of vitamin D_2_ (ergosterol) [15]. This chiral pool has been used as a starting point in the synthesis of numerous vitamin D_3_ analogues via efficient convergent strategies [18,19].

### 2.3. Synthesis of Vitamin D Analogue ***4***

**Scheme 2:**
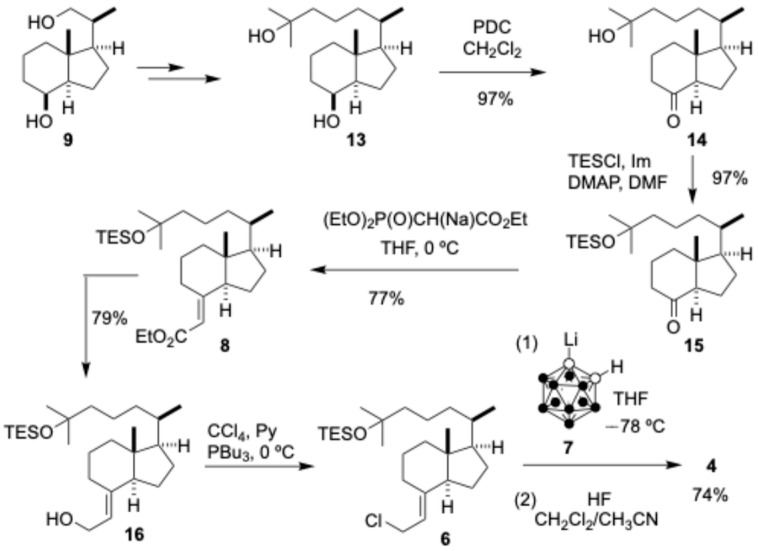
Synthesis of vitamin D_3_ analogue **4**.

Known diol **13** [20,21] (Scheme 2) was prepared from Inhoffen-Lythgoe diol (**9**) according to known procedures [22,23]. Oxidation of diol **13** with pyridinium-dichromate in CH_2_Cl_2_ afforded hydroxy-ketone **14** (97%) [24,25], which was protected with triethylsilyl chloride in the presence of imidazol, and 4-dimethylamino pyridine in DMF to provide ketone **15** (97%) [25]. Wadsworth–Horner–Emmons on ketone **15** with (EtO)_2_P(O)CH(Na)CO_2_Et, generated by reaction of (EtO)_2_P(O)CH_2_CO_2_Et with NaH in THF, gave a 3:1 mixture of (*E/Z*) esters, which could be separated by HPLC to afford **8** (77%) [25], whose reduction with LiAlH_4_ in Et_2_O provided the allylic alcohol **16** (79%) [25]. Treatment of **16** under Fuchs reaction conditions with tri-*n*-butylphosphine, pyridine, and carbon tetrachloride provided allylic chloride **6** [25], which was subjected to nucleophilic displacement with *o*-carboranyllithium (**7**) to furnish, after deprotection with 48% hydrofluoric acid in CH_2_Cl_2_/CH_3_CN, the desired carborane-vitamin D_3_ analogue **4** (74%) (two steps) (42% yield from **13** over 7 steps).

### 2.4. Synthesis of Vitamin D_3_ Analogue ***5***

**Scheme 3:**
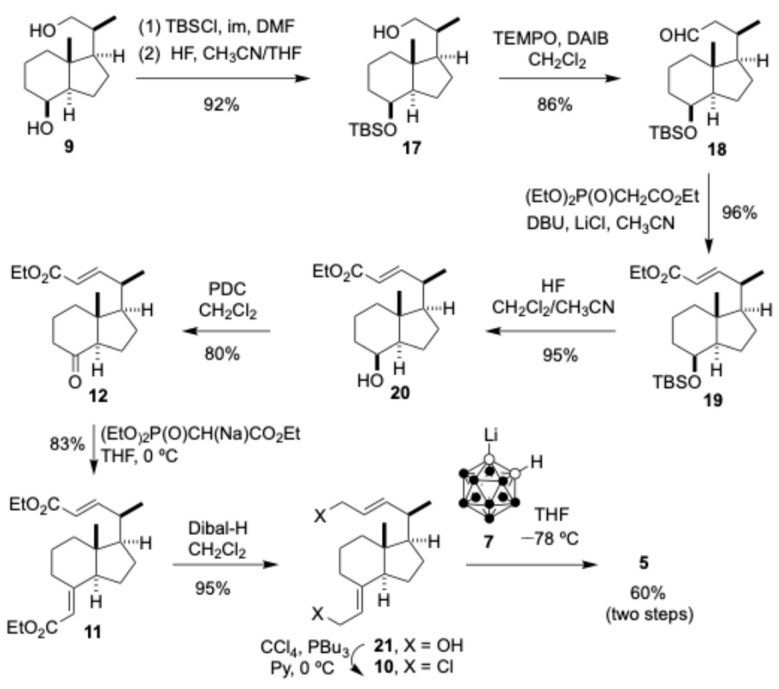
Synthesis of vitamin D analogue **5**.

The synthesis of target compound **5** also starts from Inhoffen-Lythgoe diol (**9**), which was converted to alcohol **17** [26] in 92% yield by silylation and selective deprotection as previously described [27] (Scheme 3). Oxidation of **17** with catalytic (2,2,6,6)-tetramethylpiperidine-*N*-oxyl in the presence of (diacetoxyiodo)benzene in CH_2_Cl_2_ provided the known aldehyde **18** in 86% yield [27]. Wadsworth–Horner–Emmons [28] on **18** using phosphonate (EtO)_2_P(O)CH_2_CO_2_Et in the presence of lithium chloride and 1,8-diazabicyclo[5.4.0]undec-7-ene in CH_3_CN as previously described [28] afforded, after deprotection with 48% hydrofluoric acid in CH_2_CH_2_/CH_3_CN, the α,β-unsaturated ester **20** (91%, two steps), which was oxidized with pyridinium dichromate in CH_2_Cl_2_ to give ketone **12** in 83% yield [29]. Wadsworth–Horner–Emmons on **12** with (EtO)_2_P(O)CH(Na)CO_2_Et in THF provided a (*E/Z*) mixture of α,β-unsaturated esters which was separated by HPLC to give pure **11** (83%). Diisobutylaluminum hydride reduction of **11** in CH_2_Cl_2_ furnished the allylic diol **21** in 95% yield, which was converted to the corresponding allylic dichloride **10** with carbon tetrachloride and tri-*n*-butylphosphine and pyridine. Finally, dichloride **10** was inmediately treated with carboranyllithium **7** to afford the desired vitamin D_3_ analogue **5** in 60% yield (27% from **9**, 10 steps).

### 2.5. Binding

**Figure 3:**
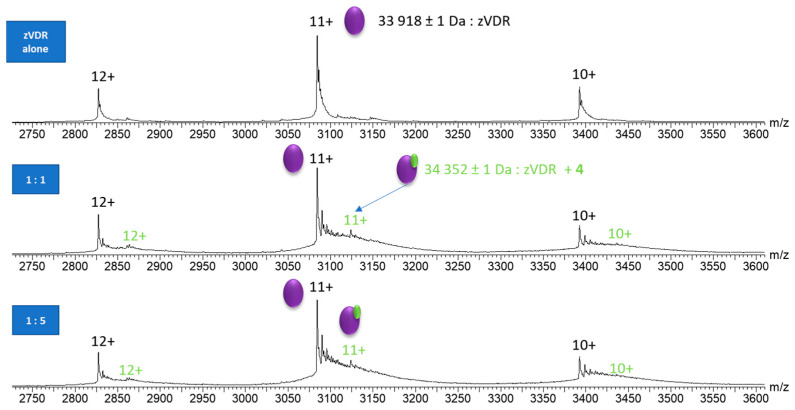
Vitamin D analogue **4** interaction with VDR. Native electrospray ionization mass spectra of zVDR LBD apo and after addition of 1, 5, and 10 molar excess of vitamin D analogue **4**.

To investigate the level of binding of the ligand **4** to zebrafish VDR ligand binding domain, we used native mass spectrometry (nMS). The ligand was added to VDR LBD at 1, 5, and 10 molar excess and the complexes were analyzed at a low voltage (40 V). In the presence of a ligand, a peak with an increment of 434 Da was observed, indicating the formation of a complex with compound **4** with a 1:1 stoichiometry (Figure 3). Increasing the molar excess of the compound does not increase the proportion of ligand bound and the main species corresponds to the apo protein. Co-crystallization assays with zVDR LBD were performed but failed.

### 2.6. Biological Activity

**Figure 4:**
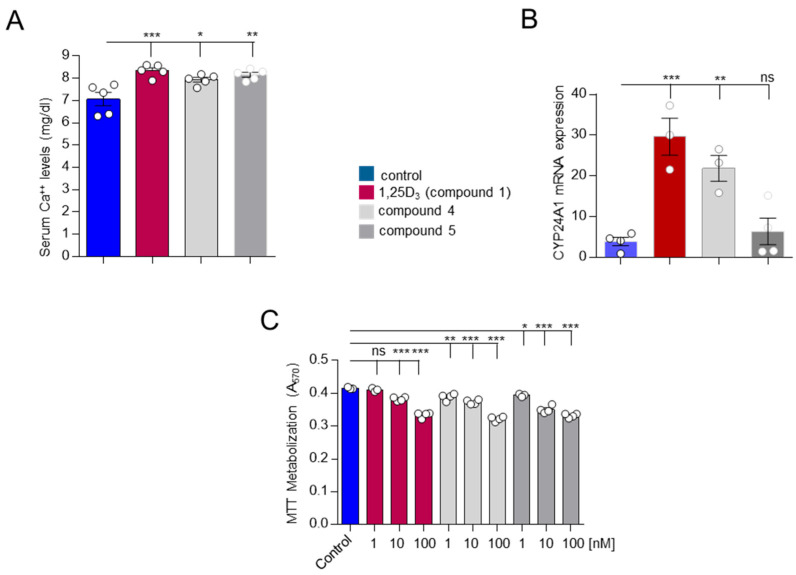
Biological properties of the vitamin D analogues. (**A**) Calcium levels in sera of mice treated intraperitoneally with sesame oil (control), 0.3 μg/kg of 1,25D_3_, and compounds **4** and **5** every other day for 21 days. (**B**) Real-time PCR of the CYP24A1 mRNA levels in mice treated for 12 h with sesame oil (control), 1,25D_3_, compound **4**, and compound **5** (0.3 μg/kg weight). (**C**) Proliferation/cytotoxicity (MTT assay) in the human breast adenocarcinoma MCF-7 cell line. Cells were treated with ethanol (control), different doses (1, 10, and 100 nM) of 1,25D_3_, and **4** and **5** analogues for 48 h, and absorbance (570 nM) was then determined. Results are expressed as mean ± SEM. * = *p* < 0.05; ** = *p* < 0.01; *** = *p* < 0.001; ns = not significant.

We analyzed the ability of vitamin D analogues **4**, and **5** to calcium mobilization. Calcium serum levels were evaluated in mice after i.p. injection of **4**, and **5** analogues, and 1,25D_3_ (Figure 4A). Our data indicated that all compounds (**4**, **5**, and 1,25D_3_) significantly raise calcium levels in relation to the vehicle-treated mice. The transcriptional activation of a vitamin D target gene CYP24A1 (24-hydroxylase, a well-known 1,25D_3_ target gene) by the analogues was evaluated by RT-PCR. Both, compound **4** and 1,25D_3_ induced the strong activation of CYP24A1 mRNA expression after 12 h of treatment (Figure 4B), but this was not the case for compound **5**. However, cell proliferation evaluated in the human breast adenocarcinoma MCF-7 cell line showed a significant (*p* < 0.001) dose-dependent decrease after 48 h of treatment with 1,25D_3_ and compounds **4** and **5** (Figure 4C). A summary of the biological results can be found in Supplementary Table SI-1.

Docking studies indicate that analogue **5** may induce steric hindrance resulting in the absence of transcriptional response, in contrast to analogue **4**. Despite this impaired binding, both compounds exhibit similar antiproliferative and calcemic effects. It is known that in addition to transcription regulation, the regulation of cell growth by vitamin D analogues can also be mediated by other important mechanisms [30].

## 3. Materials and Methods

### 3.1. Chemistry

*Ethyl (2E)-2-[(1R,3aS,7aR)-1-[(1R)-1,5-dimethyl-5-[(triethylsilyl)oxy]hexyl]octahydro-7a-methyl-4H-inden-4-ylidene]acetate* (**8**). (EtO)_2_P(O)CH_2_CO_2_Et (2.6 mL, 14.23 mmol, 11.6 equiv) was added to a 0 °C cooled suspension of NaH (0.335 g, 13.96 mmol, 11.08 equiv) in dry THF (15 mL). The mixture was stirred at room temperature for 1 h. A solution of ketone **15** (0.5 g, 1.26 mmol, 1 equiv) in THF (10 mL) was added. The reaction mixture was stirred at room temperature for 12 h. H_2_O (10 mL) and saturated NH_4_Cl (10 mL) were successively added. The mixture was extracted with TBME (3 × 25 mL). The combined organic extracts were dried (Na_2_SO_4_), filtered, and concentrated in vacuo. The residue was purified by HPLC [Phenomenex-Luna 5 µ silica (2) column, Ø 10 × 250 mm, 8% EtOAc/hexanes] to give pure **8** (0.451 g, 77%, colourless oil, *Rf* = 0.73 (15% EtOAc/hexane). ${\left[\alpha \right]}_{D}^{25}$ = 89.2 (c = 2.4 CHCl_3_). **^1^H NMR** (250 MHz, CDCl_3_): δ 5.45 (1H, s, HC=), 4.14 (2H, q, *J* = 7.1 Hz, O**CH_2_**CH_3_), 3.85 (1H, m, CH), 1.28 (3H, t, *J* = 7.2 Hz, OCH_2_**CH_3_**), 1.18 (6H, s, CH_3_-6′ and CH_3_-7′), 0.97–0.90 [12H, m, CH_3_-1′, (**CH_3_**CH_2_)_3_Si], 0.60-0.50 [9H, m, CH_3_-8, (CH_3_**CH_2_**)_3_Si]. **^13^C NMR** (63 MHz, CDCl_3_): δ 166.8 (C, C=O), 163.4 (C, C-4), 111.7 (CH, HC=), 73.3 (C, C-6′), 59.3 (CH_2_), 56.7 (CH), 29.6 (CH), 56.6 (CH), 46.9 (C, C-7a), 45.3 (CH_2_), 40.0 (CH_2_), 36.2 (CH_2_), 35.8 (CH), 29.8 (CH), 29.6 (CH), 29.5 (CH_2_), 27.3 (CH), 23.7 (CH_2_), 22.0 (CH_2_), 20.6 (CH_2_), 18.6 (CH_3_, C-1′), 14.2 (CH), 11.9 (CH_3_, C-8), 6.9 [(**C**H_3_CH_2_)_3_Si], 6.6 [(CH_3_**C**H_2_)_3_Si]. **IR** (film, cm^−1^): 2950, 2909, 2874, 2732, 1715. **HRMS**: [ESI]^+^, cald for [C_28_H_52_O_3_NaSi]^+^, [M + Na]_+_: 487.3577; found: 487.3577.

*2-[(1R,3aS,7aR)-1-[(1R)-1,5-Dimethyl-5-[(triethylsilyl)oxy]hexyl]octahydro-7a-methyl-4H-inden-4-ylidene]-Ethanol* (**16**). LiAlH_4_ (0.047 g, 1.23 mmol, 2 equiv) was added to a 0 °C cooled solution of **8** (0.25 g, 0.618 mmol, 1 equiv) in dry THF (15 mL). The reaction mixture was stirred for 3 h. H_2_O (2 mL) was added dropwise. The mixture was stirred for 10 min and then extracted with EtOAc (3 × 15 mL). The combined organic extracts were dried, filtered, and concentrated in vacuo. The residue was purified by flash column chromatography (SiO_2_, Ø 2.5 × 8 cm, 10% EtOAc/hexanes) to give **16** [0.205 g, 79%, colourless oil, *Rf* = 0.33 (10% EtOAc/hexanes), ${\left[\alpha \right]}_{D}^{25}$ = 62.7 (c = 3.0, CHCl_3_)]. **^1^H NMR** (250 MHz, CDCl_3_): δ 5.21 (1H, t, *J* = 7.1 Hz, H-2), 4.20 (2H, d, *J* = 7.1 Hz, H-1), 2.62 (1H, d, *J* = 12.1 Hz), 1.18 (6H, s, CH_3_-6″ and CH_3_-7″), 0.97-0.90 [12H, m, CH_3_-1″, (**CH_3_**CH_2_)_3_Si], 0.60-0.50 [9H, m, CH_3_-8′, (CH_3_**CH_2_**)_3_Si]. **^13^C NMR** (63 MHz, CDCl_3_): δ 143.6 (C, C-4′), 118. 9 (CH, C-2), 73.3 (C, C-6″), 58.5 (CH_2_, C-1), 56.4 (CH), 55.5 (CH), 45.3 (CH_2_), 45.2 (C, C-7a′), 40.2 (CH_2_), 36.3 (CH_2_), 35.9 (CH), 29.8 (CH), 29.6 (CH), 28.6 (CH_2_), 27.5 (CH), 23.4 (CH_2_), 22.0 (CH_2_), 20.6 (CH_2_), 18.6 (CH_3_, C-1″), 11.7 (CH_3_, C-8′), 6.9 [(**CH_3_**CH_2_)_3_Si], 6.6 [(CH_3_**CH_2_**)_3_Si]. **IR** (film, cm^−1^): 3339, 2950, 2927, 2874. **HRMS**: [ESI-TOF]^+^, calcd for [C_26_H_50_O_2_NaSi]^+^, [M + Na]^+^: 445.3472; found: 445.3481.

*(6R)-2-Methyl-6-((3aS,7aR,E)-7a-methyl-4-(2-o-carboranylethylidene)octahydro-1H-inden-1-yl)heptan-2-ol* (**4**). A solution of *n*-BuLi in hexanes (0.549 mL, 1.43 M, 0.785 mmol, 2.1 equiv) was added dropwise to a −78 °C cooled solution of *o*-carborane (0.108, 0.748 mmol, 2 equiv) in dry THF (7 mL). The reaction mixture was stirred for 1 h. A solution of freshly prepared chloride **6** (0.165 g, 0.374 mmol, 1 equiv) in dry THF (5 mL) was added via cannula. The reaction mixture was stirred at room temperature for 1 h. H_2_O (3 mL) was added. The mixture was extracted wit TBME (2 × 10 mL). The combined organic extracts were dried, filtered, and concentrated in vacuo. The residue was dissolved in a mixture CH_2_Cl_2_ (4 mL) and CH_3_CN (8 mL). HF (48%, 15 drops) were added. The mixture was stirred for 2 h. The reaction mixture was poured into saturated NaHCO_3_ (50 mL). The mixture was extracted CH_2_Cl_2_ (3 × 10 mL). The combined organic extracts were dried, filtered, and concentrated in vacuo. The residue was flash chromatographed (SiO_2_, Ø 1.5 × 5 cm, 20% EtOAc/hexanes) to give the vitamin D_3_ analogue **4** [0.120 g, 74%, *Rf* = 0.23 (20% EtOAc/hexanes, white foam]. ${\left[\alpha \right]}_{D}^{25}$ = 50.9 (c = 2.6, CHCl_3_). **^1^H NMR** (500 MHz, CDCl_3_) (Steroidal numbering): δ 4.85 (1H, t, *J* = 8.1 Hz, H-7), 3.55 (1H, s, H-carb), 2.99 (2H, qd, *J* = 14.9 Hz, *J* = 8.1 Hz), 2.44 (1H, d, *J* = 10.5 Hz), 1.21 (6H, s, CH_3_-26 and CH_3_-27), 0.94 (3H, d, *J* = 6.2 Hz, CH_3_-21), 0.53 (3H, s, CH_3_-18). **^13^C NMR** (63 MHz, CDCl_3_): δ 145.6 (C, C-8), 112.9 (CH, C-7), 75.0 (C, Carb), 70.9 (C, HC-carb), 56.3 (CH), 55.5 (CH), 45.3 (CH_2_), 44.2 (C, C-13), 39.9 (CH_2_), 36.2 (CH_2_), 35.9 (CH), 34.7 (CH_2_), 29.3 (CH), 29.1 (CH), 28.5 (CH_2_), 27.4 (CH_2_), 23.3 (CH_2_), 22.1 (CH_2_), 20.6 (CH_2_), 18.6 (CH_3_, C-21), 11.8 (CH_3_, C-18). **^11^B NMR** (160.46 MHz, CDCl_3_): δ −3.38, −6.87, −10.15, −11.88, −14.19. **UV** (96% EtOH): λ max = 292 nm (ε = 1.000), λ = 206 nm (ε = 10,000). **IR** (film, cm^−1^): 3378, 2931, 2869, 2581. **HRMS**: [ESI-TOF]^−^, cald for [C_22_H_45_B_10_O]^+^, [M-H]^+^: 435.4440; found: 435.4435.

*Ethyl (R,E)-4-((1R,3aS,7aR,E)-4-(2-ethoxy-2-oxoethylidene)-7a-methyloctahydro-1H-inden-1-yl)pent-2-enoate* (**11**). (EtO)_2_P(O)CH_2_CO_2_Et (2.08 mL, 9.3 mmol, 19.05 equiv) was slowly added to a 0 °C cooled suspension of NaH (0.213 g, 8.7 mmol, 17.83 equiv) in dry THF (10 mL). The mixture was stirred at room temperature for 1 h. A solution of ketone **12** (0.136 g, 0.488 mmol, 1 equiv) in dry THF (5 mL) was added via cannula. The reaction mixture was stirred for 12 h. H_2_O (10 mL) and saturated NH_4_Cl (10 mL) were successively added. The mixture was extracted with TBME (3 × 25 mL). The combined organic extracts were dried, filtered, and concentrated in vacuo. The residue was purified by HPLC (Phenomenex-LUNA 5 µ Silica (2), Ø 10 × 250 mm, 2% EtOAc/hexanes) to give pure **11** [0.136 g, 83%, colourless oil, *Rf* = 0.66 (15% EtOAc/hexanes)]. **^1^H NMR** (250 MHz, CDCl_3_): δ 6.74 (1H, dd, *J* = 15.6 Hz, *J* = 9.0 Hz, H-3), 5.72 (1H, d, *J* = 15.5 Hz, H-2), 5.42 (1H, d, *J* = 1.9 Hz, H-1″), 4.14 (4H, q, *J* = 8.5 Hz, 2 × (O**CH_2_**CH_3_), 3.83 (1H, m), 2.42 (1H, m), 2.08 (1H, t, *J* = 9.4 Hz), 1.25 (6H, t, *J* = 8.5 Hz, 2 × (OCH_2_**CH_3_**), 1.07 (3H, dd, *J* = 6.5 Hz, *J* = 1.8 Hz, H- 5), 0.57 (3H, s, CH_3_-8′). **^13^C NMR** (63 MHz, CDCl_3_): δ 166.7 (C, C=O), 166.6 (C, C=O), 162.4 (C, C-4′), 153.8 (C, C-3), 119.2 (CH, C-2), 112.1 (CH, C-1′), 59.9 (CH_2_) 59.3 (CH_2_), 56.4 (CH), 52.3 (CH), 46.9 (C, C-7a′), 39.7 (CH_2_), 39.6 (CH), 29.3 (CH_2_), 27.1 (CH_2_), 23.5 (CH_2_), 21.9 (CH_2_), 19.2 (CH_3_), 18.9 (CH_2_), 14.1 (CH_3_, C-5), 12.2 (CH_3_, C-8′).

*(1R,3aS,7aR,E)-7a-Methyl-4-(2-o-carboranyl-ethylidene)-1-((R,E)-5-o-carboranyl-lpent-3-en-2-yl)octahydro-1H-indene* (**5**). A solution of *n*-BuLi in hexanes (0.958 mL, 1.37 mmol, 1.43 M, 4.1 equiv) was added dropwise to a −78 °C cooled solution of *o*-carborane (0.193 g, 1.34 mmol, 4 equiv) in dry THF (10 mL). The reaction mixture was stirred at room temperature for 1 h. A solution of fresh dichloride **10** (0.165 g, 0.374 mmol, 1 equiv) in dry THF was added via cannula. The reaction mixture was stirred at room temperature for 1 h. H_2_O (4 mL) was added. The mixture was extracted with TBME (3 × 20 mL). The combined organic extracts were dried, filtered, and concentrated in vacuo. The residue was flash chromatographed (SiO_2_, Ø 2.5 × 7 cm, 5% EtOAc/hexanes) to give vitamin D analogue **5** [0.101 g, 60%, white foam, *Rf* = 0.66 (10% EtOAc/hexanes)]. **^1^H NMR** (500 MHz, CDCl_3_): δ 5.39 (1H, dd, *J* = 15.1 Hz, *J* = 8.6 Hz), 5.24 (1H, dt, *J* = 15.0 Hz, *J* = 7.3 Hz), 4.86 (1H, t, *J* = 8.1 Hz), 3.54 (2H, s, HC-carb), 2.99 (3H, qd, *J* = 15.0 Hz, *J* = 8.1 Hz), 2.86 (2H, d, *J* = 7.3 Hz), 2.45 (2H, d, *J* = 11.3 Hz), 1.03 (3H, CH_3_-5), 0.55 (3H, s, CH_3_-8′). **^13^C NMR** (63 MHz, CDCl_3_): δ 145.1 (C, C-4′), 143.2 (CH), 120.5 (CH), 113.2 (CH), 79.9 (C), 74.4 (C), 59.3 (CH), 55.5 (CH), 55.4 (CH), 45.3 (C, C-7a′), 40.6 (CH_2_), 40.1 (CH), 39.8 (CH_2_), 34.7 (CH_2_), 28.4 (CH_2_), 27.6 (CH_2_) 23.2 (CH_2_), 22.1 (CH_2_), 20.3 (CH_3_, C-5), 12.0 (CH_3_, C-8′). **^11^B NMR** (160.46 MHz, CDCl_3_): δ −3.42, −6.81, −10.17, −12.01, −14.08. **UV** (96% EtOH): λ max = 292 nm (ε = 2800), λ = 206 nm (ε = 20,000). **IR** (film, cm^−1^): 2948, 2869, 2588. **HRMS** [ESI-TOF]^−^, calcd for [C_21_H_47_B_20_]^−^, [M-H]^−^: 519.5598; found: 519.5547.

### 3.2. Binding

#### 3.2.1. Protein Production and Purification

The cDNA encoding His-tagged zVDR LBD (156–453) was cloned into pET28b. The recombinant proteins were produced in Escherichia coli BL21 DE3 strain grown for 4 h at 20 °C after induction with 1 mM IPTG at an OD600 of ~0.7. Soluble proteins were purified on Ni Hitrap FFcrude column (Cytiva, Upsalla, Sweden), followed by size exclusion chromatography on HiLoad Superdex 75 column (Cytiva) equilibrated in Tris 20 mM pH7, NaCl 200 mM, TCEP 1 mM. In some experiments, the His tag was removed by thrombin cleavage before size exclusion chromatography.

#### 3.2.2. Mass Spectrometry Analysis

Prior to mass spectrometry analysis in native condition, VDR LBDs were buffer exchanged against 200 mM of ammonium acetate at pH 6.9, using microspin Zeba desalting coulumn (cutoff 7 kDa). The samples were diluted in 200 mM AcONH_4_ to a final concentration of 10 µM and infused in triplicates with an automated chip based nanoelectrospray device (Triversa Nanomate, Advion Bioscience, Ithaca, NY, USA) operating in the positive ion mode, coupled to a Synapt G2 HDMS mass spectrometer (Waters, Manchester, UK). The cone voltage and the backing pressure of the mass spectrometer were set to 40 V, and 6 mbar, respectively, to avoid protein/ligand dissociation.

### 3.3. Biological Assays

#### 3.3.1. Serum Calcium Evaluation

All animal studies were approved by the University of Santiago de Compostela Ethics Committee for Animal Experiments. The 1,25D_3_ and compounds **4** and **5** (0.3 μg/kg weight) were injected intraperitoneally (i.p.) into groups of 5 male Swiss cd-1 mice. The injections were performed every other day for 21 days using sesame oil as a vehicle. At the end of the test, serum calcium was determined using the QuantiChom calcium assay kit (BioAssay Systems, Hayward, CA, USA).

#### 3.3.2. Real-Time PCR

Twelve hours after last treatment (see serum calcium evaluation), a piece of tail tissue from mice was obtained, total RNA was isolated using TRIzol reagent (Invitrogen, Barcelona, Spain), and the cDNA synthesis was carried out using 1 μg of total RNA. cDNA was used for CYP24A1 (24-hydroxylase) quantification by real-time PCR (Eppendorf Master cycler ep realplex, Hamburg, Germany) using Luminaris Color HiGreen qPCR Master Mix (Thermo Fisher Scientific, Barcelona, Spain) as previously described [31].

#### 3.3.3. Cell Proliferation/Cytotoxicity

The human breast adenocarcinoma MCF-7 cell line was obtained from ATCC-LGC (Barcelona, Spain), and grown as previously described [31]. MCF-7 cells were treated with 1,25D_3_. and the compounds **4** and **5** at 10^−9^ to 10^−7^ M for 48 h, and an MTT (Merck, Darmstadt, Germany) assay was performed. Absorbance was measured at 570 nm in a Mithras LB 940 from Berthold Technologies (Bad Wildbad, Germany).

## 4. Conclusions

In summary, we designed and synthesized vitamin D analogues **4** and **5** of the hormone 1α,25-dihydroxyvitamin D_3_. Both analogues contain an *o*-carboranyl unit replacing the A-ring, but analogue **5** contains an additional *ortho*-carboranyl unit at the side chain. Vitamin D analogues **4** and **5** were synthesized from Inhoffen-Lythgoe diol in 25% overall yield (11 steps) and 27% yield (9 steps), respectively. Highlights of the synthetic route include the *S_N_2*-displacement of allylic chlorides by *ortho*-carboranyl lithium. Possible improvements in the synthesis of **5** include the preparation of compound **20** from the Inhoffen-Lythgoe diol without using protecting groups. Docking and binding assay indicate that the carboranyl-A-ring is accommodated in the active binding pocket. Biological data indicate that both compounds are VDR agonists, and like 1,25D_3_, are hypercalcemic. Both analogues also significantly decrease cell proliferation in tumour cells, although only compound **4** increases the mRNA expression of CYP24A1, a target gene of 1,25D_3_. Although in this study only intraperitoneal administration of both analogues (**4**, and **5**) were used in vivo, it would be interesting to study their pharmacokinetic properties, evaluating other routes of administration, such as oral administration, as well as their distribution and metabolization.

## Data Availability

The original contributions presented in this study are included in the article. Further inquiries can be directed to the corresponding author(s).

## Supplementary Materials

The following supporting information can be downloaded at: https://www.mdpi.com/article/10.3390/molecules30122637/s1, ^1^H NMR spectra and ^13^C NMR of compounds (pp: S3–S16), Figure SI-1: Superimposition compound **4** and 1,25D_3_ (p: S17), summary of the biological data (p: S17); Table SI-1: Serum calcium levels in mice, mRNA expression of the gene encoding CYP24A1, and cytotoxicity (MTT assay) of 1,25D_3_, compound **4** and compound **5**.
