# Supplementary material for: Synthesis, Molecular Docking and Biological Evaluation of A-Ring-Carborane-Vitamin D Analogues
Source: Molecules. 2025 Jun 18;30(12):2637. doi: 10.3390/molecules30122637 (PMC12195832; doi:10.3390/molecules30122637)
Supplement: Supplementary file 1 [file molecules-30-02637-s001.zip › molecules-3692092-supplementary.pdf]

Supplementary material

Synthesis, Molecular Docking and Biological Evaluation of A-Ring-Carborane-Vitamin D Analogues

Rocío Otero,[1] Samuel Seoane,[2] Xoán Fernández-Domínguez,[1] Maxime Bourguet,[3,4] Sarah Cianférani,[3,4] Carole Peluso-Iltis,[5] Miguel A. Maestro,[6] Román Pérez-Fernández,[2] Natacha Rochel,[5] Antonio Mouriño[1]

[1] Ignacio Ribas Research Laboratory, Department of Organic Chemistry, University of Santiago de Compostela, 15782 Santiago de Compostela, Spain
[2] Department of Physiology-Center for Research in Molecular Medicine and Chronic Diseases (CIMUS), University of Santiago de Compostela, 15707 Santiago de Compostela, Spain
[3] Laboratoire de Spectrométrie de Masse BioOrganique, Université de Strasbourg, CNRS, IPHC UMR 7178, 67037 Strasbourg, France
[4] Infrastructure Nationale de Protéomique ProFI-UAR2048, 67087 Strasbourg, France
[5] Institut de Génétique et de Biologie Moléculaire et Cellulaire (IGBMC); Université de Strasbourg, CNRS, Inserm, UMR 7104- UMR-S 1258, 67400 Strasbourg, France
[6] Departamento de Química-CICA, Universidad de A Coruña, 15071 A Coruña, Spain

^{1}H and ^{13}C NMR……………………………………………………………..S3-S16

Figure SI-1. Superimposition Compound 4 and 1,25D$_3$…….……...……..…..S17

Table S1. Summary of the biological results……….………………………..S17

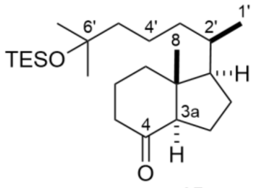

15

^{1}H NMR (250 MHz, $CDCl_3$): δ 1.14 (6H, s, CH_3-6' y CH_3-7'), 0.94-0.84 [12H, m, CH_3-1', $(C\underline{H}_3CH_2)_3Si$], 0.61-0.45 [9H, m, CH_3-8, $(CH_3C\underline{H}_2)_3Si$].

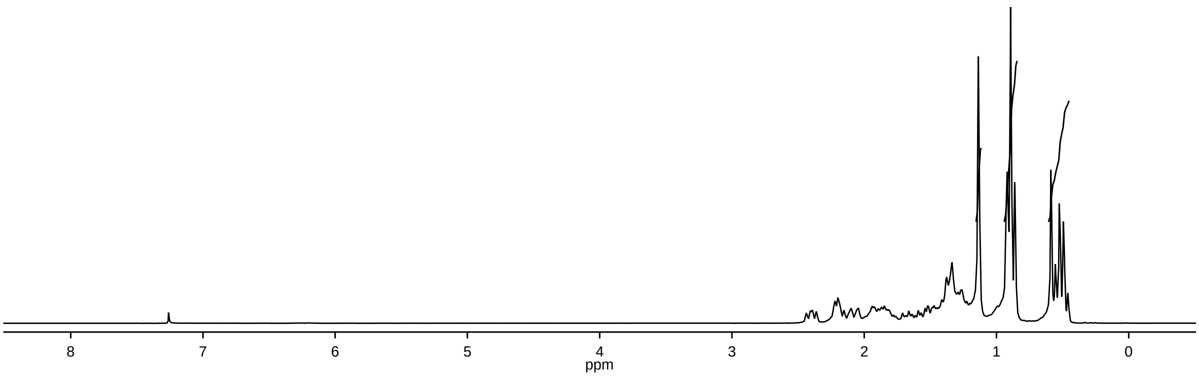

^{13}C NMR (63 MHz, $CDCl_3$): δ 211.9 (C, C-4), 73.2 (C, C-6'), 61.8 (CH, C-3a), 56.5 (CH, C-1), 49.7 (C, C-7a), 45.2 (CH_2, C-5'), 40.8 (CH_2), 38.8 (CH_2), 36.1 (CH_2), 35.3 (CH, C-1'), 29.8 (CH_3, C-6'), 29.6 (CH_3, C-7'), 27.3 (CH_2), 23.9 (CH_2), 20.5 (CH_2), 18.9 (CH_2), 18.5 (CH_3, C-1'), 12.3 (CH_3, C-8), 6.9 [$(\underline{C}H_3CH_2)_3Si$], 6.6 [$(CH_3\underline{C}H_2)_3Si)$].

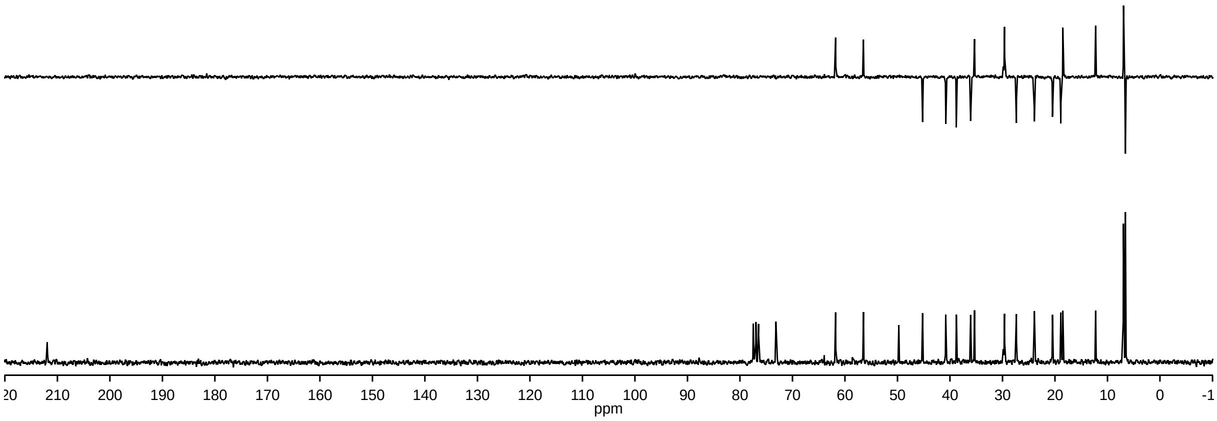
20
210
200
190
180
170
160
150
140
130
120
110
100
90
80
70
60
50
40
30
20
10
0
-1
ppm

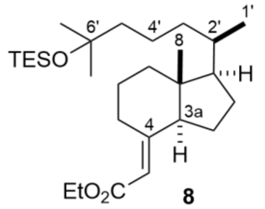

8

^{1}H NMR (250 MHz, $CDCl_3$): δ 5.45 (1H, s, HC_{sp^2}), 4.14 (2H, q, J = 7.1 Hz, $OC\underline{H}_2CH_3$), 3.85 (1H, m, CH), 1.28 (3H, t, J = 7.2 Hz, $OCH_2C\underline{H}_3$), 1.18 (6H, s, CH_3-6' y CH_3-7'), 0.97-0.90 [12H, m, CH_3-1', $(C\underline{H}_3CH_2)_3Si$], 0.60-0.50 [9H, m, CH_3-8, $(CH_3C\underline{H}_2)_3Si$].

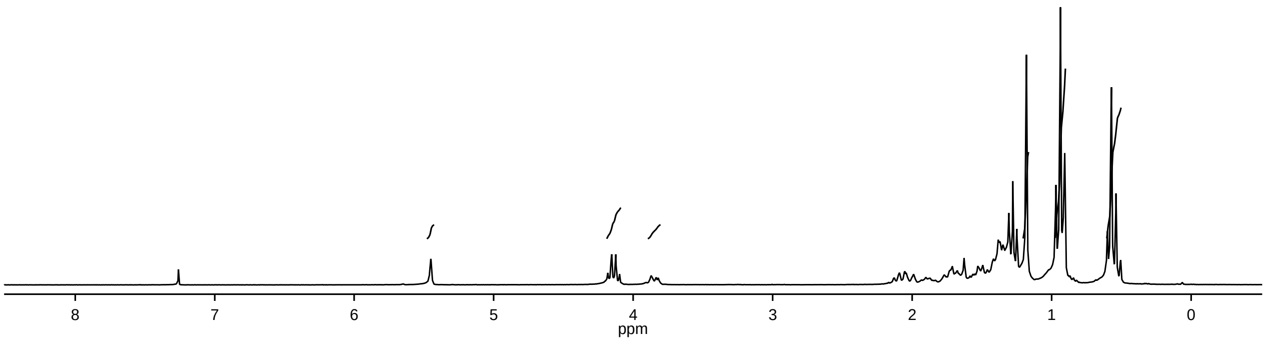

^{13}C NMR (63 MHz, $CDCl_3$): δ 166.8 (C, C=O), 163.4 (C, C-4), 111.7 (CH, HC_{sp^2}), 73.3 (C, C-6'), 59.3 (CH_2), 56.7 (CH), 56.6 (CH), 46.9 (C, C-7a), 45.3 (CH_2), 40.0 (CH_2), 36.2 (CH_2), 35.8 (CH), 29.8 (CH), 29.6 (CH), 29.5 (CH_2), 27.3 (CH), 23.7(CH_2), 22.0 (CH_2), 20.6 (CH_2), 18.6 (CH_3, C-1'), 14.2 (CH), 11.9 (CH_3, C-8), 6.9 [$(\underline{C}H_3CH_2)_3Si$], 6.6 [$(CH_3\underline{C}H_2)_3Si$].

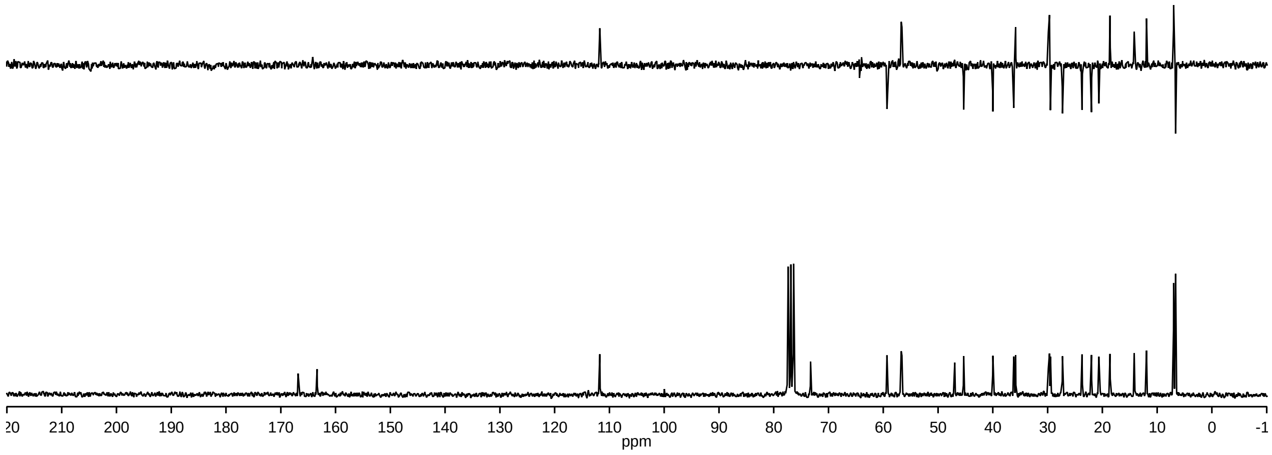

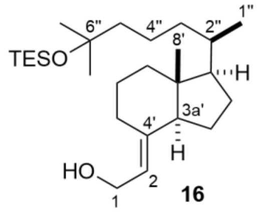

16

^{1}H NMR (250 MHz, $CDCl_3$): δ 5.21 (1H, t, *J* = 7.1 Hz, H-2), 4.20 (2H, d, *J* = 7.1 Hz, H-1), 2.62 (1H, d, *J* = 12.1 Hz), 1.18 (6H, s, CH_3-6'' y CH_3-7''), 0.97-0.90 [12H, m, CH_3-1'', $(C\underline{H}_3CH_2)_3Si$], 0.60-0.50 [9H, m, CH_3-8', $(CH_3C\underline{H}_2)_3Si$].

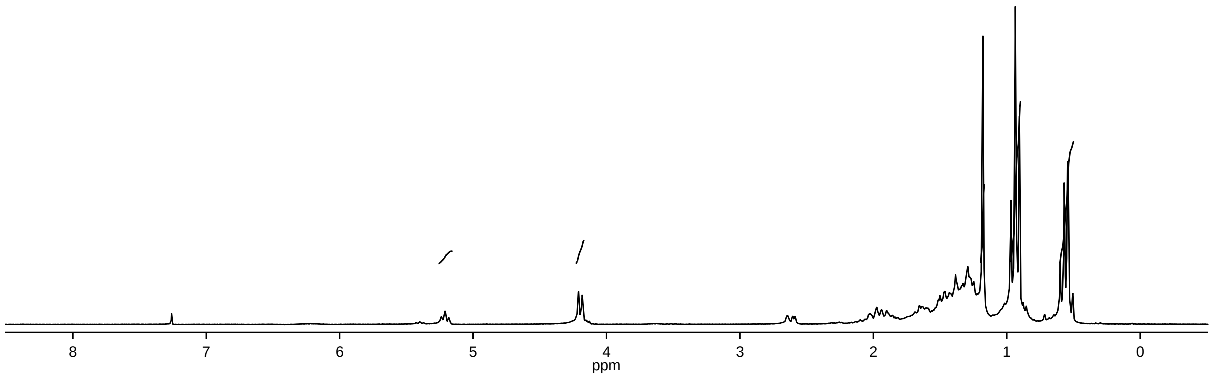

^{13}C NMR (63 MHz, $CDCl_3$): δ 143.6 (C, C-4'), 118.9 (CH, C-2), 73.3 (C, C-6''), 58.5 (CH_2, C-1), 56.4 (CH), 55.5 (CH), 45.3 (CH_2), 45.2 (C, C-7a'), 40.2 (CH_2), 36.3 (CH_2), 35.9 (CH), 29.8 (CH), 29.6 (CH), 28.6 (CH_2), 27.5 (CH), 23.4 (CH_2), 22.0 (CH_2), 20.6 (CH_2), 18.6 (CH_3, C-1''), 11.7 (CH_3, C-8'), 6.9 [$(\underline{C}H_3CH_2)_3Si$], 6.6 [$(CH_3\underline{C}H_2)_3Si$].

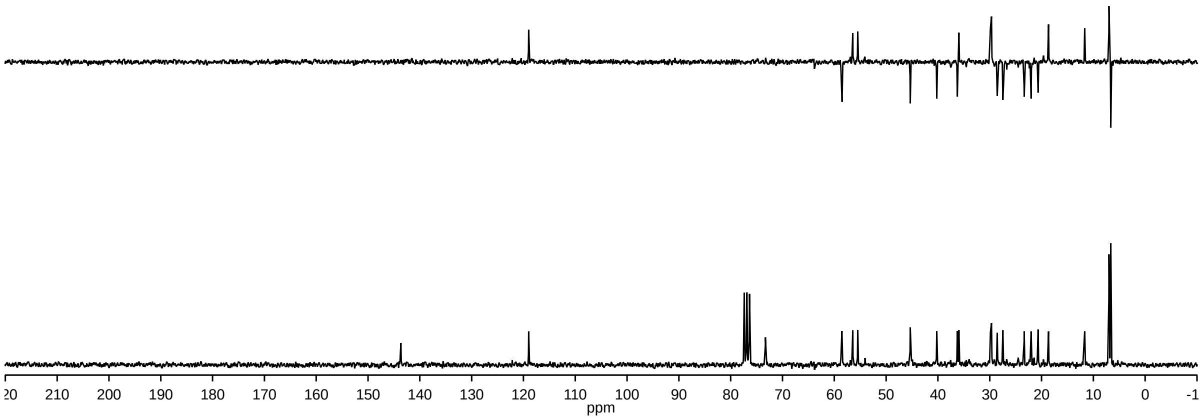

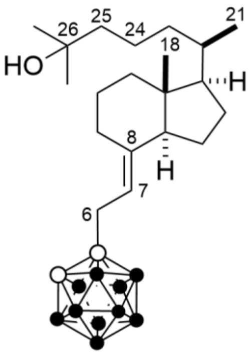

4

^{1}H NMR (500 MHz, $CDCl_3$): δ 4.85 (1H, t, *J* = 8.1 Hz, H-7), 3.55 (1H, s, H-C-Carb), 2.99 (2H, cd, *J* = 14.9 Hz, *J* = 8.1 Hz), 2.44 (1H, d, *J* = 10.5 Hz), 1.21 (6H, s, CH_3-26 y CH_3-27), 0.94 (3H, d, *J* = 6.2 Hz, CH_3-21), 0.53 (3H, s, CH_3-18).

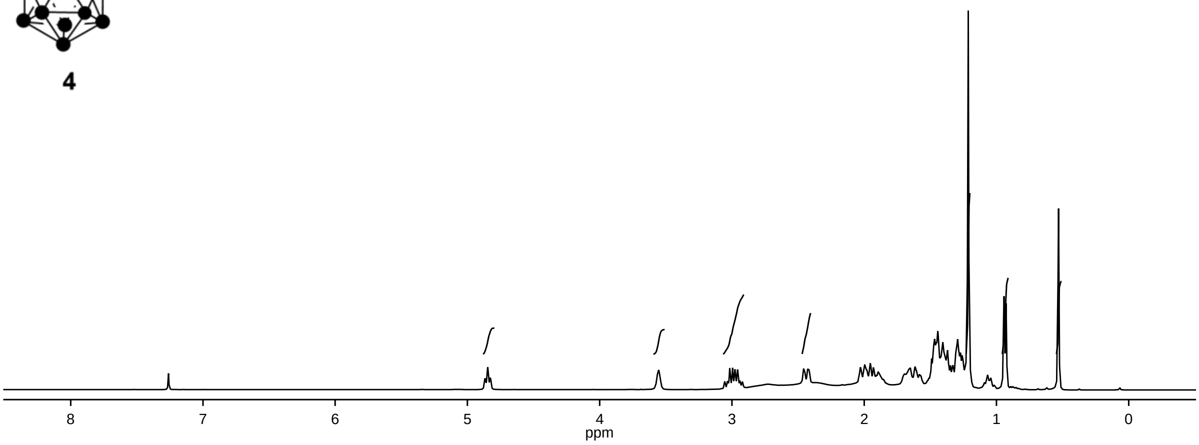

^{13}C NMR (63 MHz, $CDCl_3$): δ 145.6 (C, C-8), 112.9 (CH, C-7), 75.0 (C, C-Carb), 70.9 (C, C-2), 59.2 (CH, HC-Carb), 56.3 (CH), 55.5 (CH), 45.3 (CH_2), 44.2 (C, C-13), 39.9 (CH_2), 36.2 (CH_2), 35.9 (CH), 34.7 (CH_2), 29.3 (CH), 29.1 (CH), 28.5 (CH_2), 27.4 (CH_2), 23.3 (CH_2), 22.1 (CH_2), 20.6 (CH_2), 18.6 (CH_3, C-21), 11.8 (CH_3, C-18).

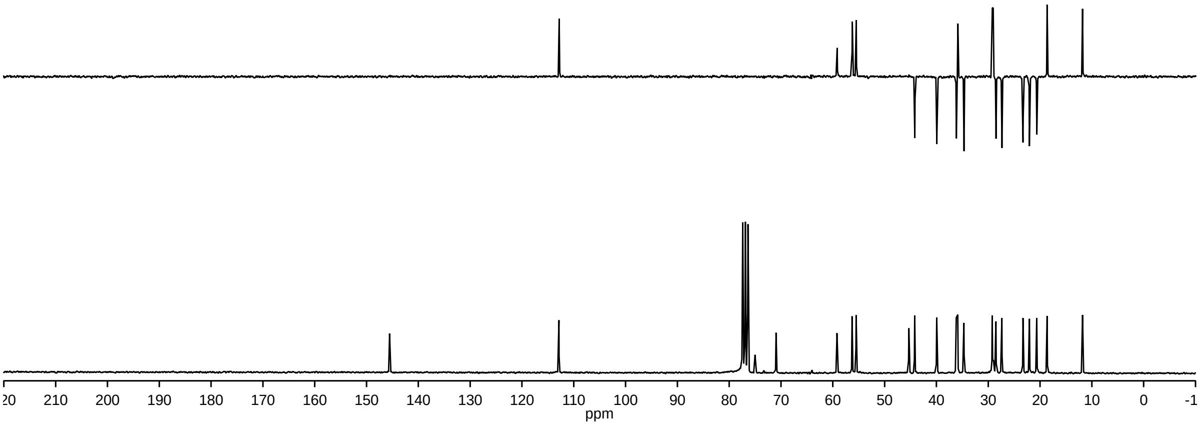

^{11}B NMRc (160.46 MHz, $CDCl_3$): δ -2.96, -3.91, -6.46, -7.25, -9.66, -10.59, -11.27, -12.26, -13.51.

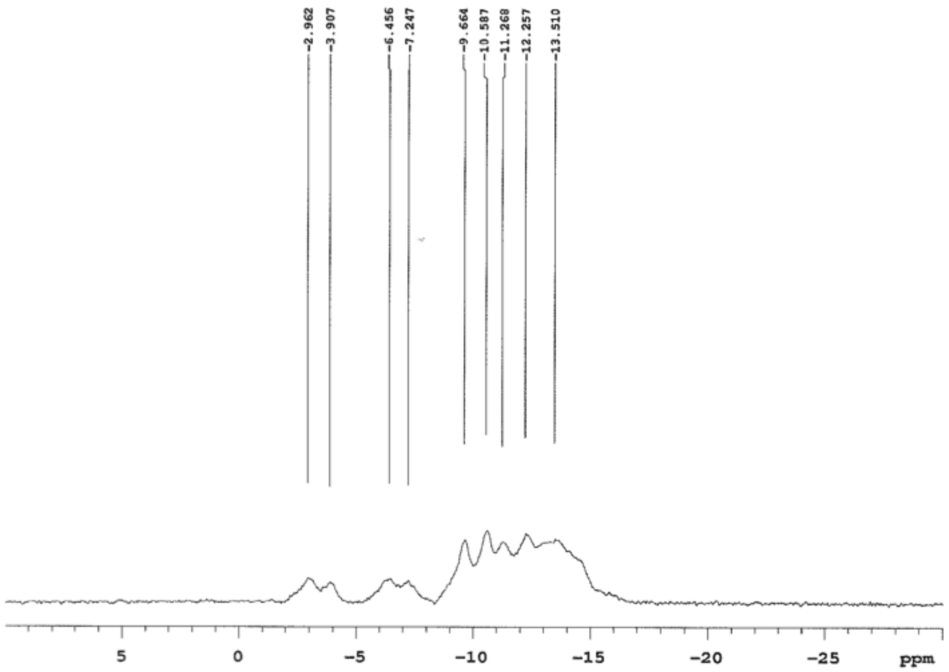

^{11}B NMRdec (160.46 MHz, $CDCl_3$): δ -3.38 -6.87, -10.15, -11.88, -14.19.

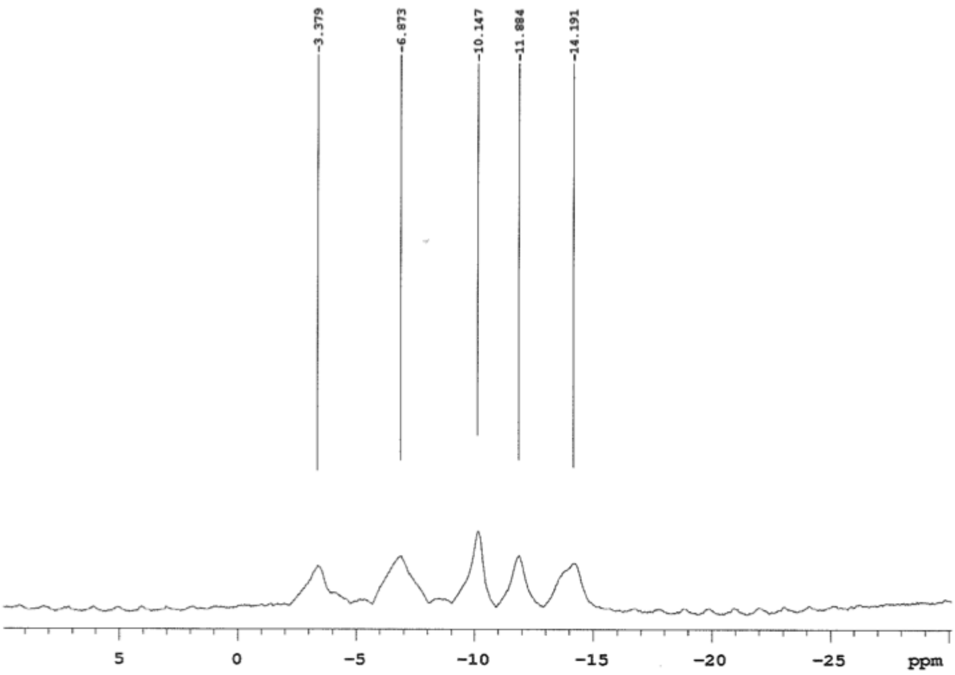

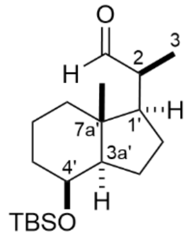

18

^{1}H NMR (250 MHz): δ 9.56 (1H, d, *J* = 3.3 Hz, H-1), 4.01 (1H, s, H-4'), 2.34 (2H, m), 1.08 (3H, d, *J* = 6.9 Hz, CH_3-3), 0.95 (3H, s, CH_3-8'), 0.87 (9H, s, Me_3C-Si), -0.01 (6H, 2xMe-Si).

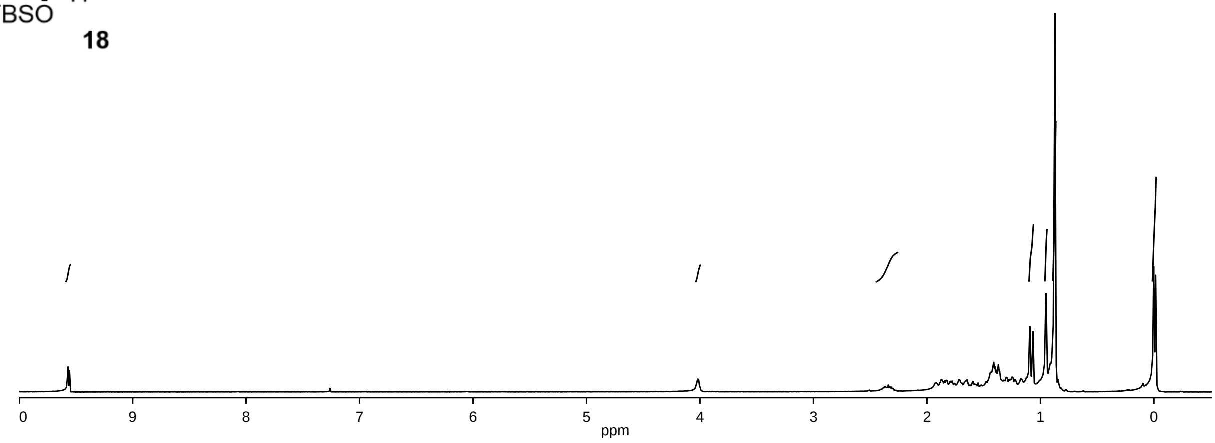

^{13}C NMR ($CDCl_3$, 63 MHz): δ 205.7 (C, CHO), 69.5 (CH, C-4'), 52.7 (CH), 52.1 (CH), 49.6 (CH), 43.0 (C, C-7a'), 40.8 (CH_2), 34.7 (CH_2), 26.6 (CH_2), 26.2 (3xCH_3, Me_3CSi), 23.7 (CH_2), 18.4 (C, Me_3CSi), 17.9 (CH_2), 14.5 (CH_3, C-3), 13.7 (CH_3, C-8'), -4.4 (CH_3, MeSi), -4.7 (CH_3, MeSi.

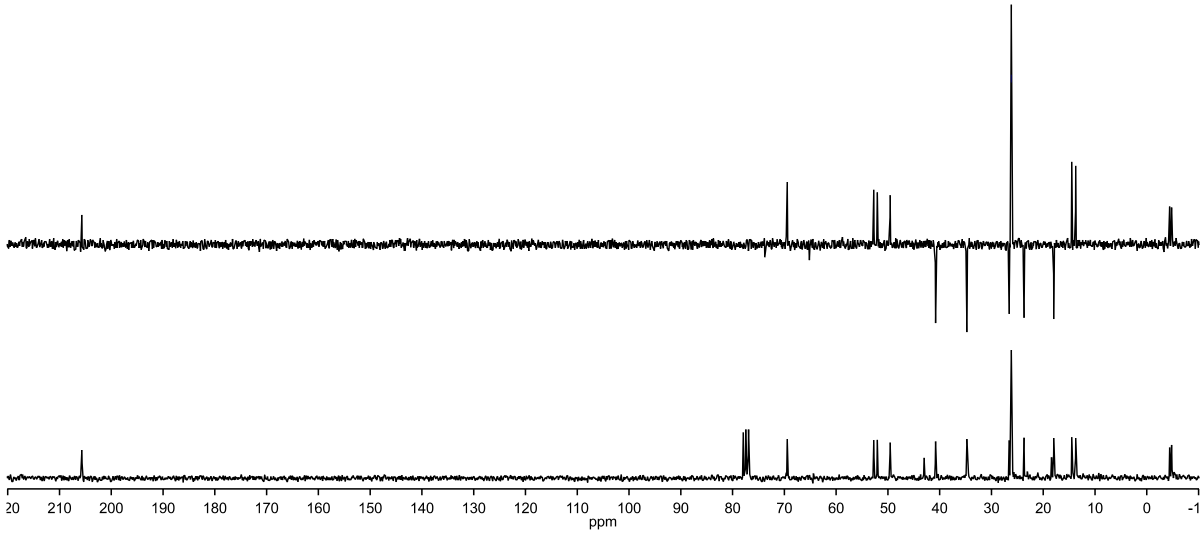

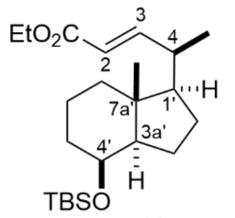

19

^{1}H NMR (250 MHz, $CDCl_3$): δ 6.76 (1H, dd, *J* = 15.6 Hz, *J* = 8.8 Hz, H-3) 5.66 (1H, d, *J* = 15.5 Hz, H-2), 4.06 (2H, c, *J* = 7.1 Hz, OC$\underline{H}_2$CH$_3$), 3.94 (1H, s, H-4'), 1.25 (3H, t, *J* = 7.2 Hz Hz, OCH$_2$C$\underline{H}_3$), 0.99 (3H, d, *J* = 6.5 Hz, H-5), 0.89 (3H, s, CH$_3$-8'), 0.82 (9H, s, Me$_3$CSi), -0.06 (6H, d, *J* = 4.1 Hz, Me$_2$Si).

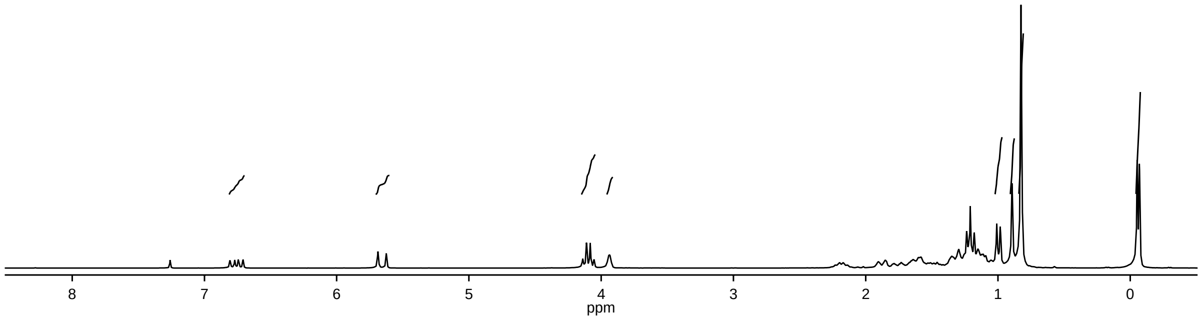

^{13}C NMR (63 MHz, $CDCl_3$): δ 166.7 (C, C=O), 154.5 (C, C-3), 118.7 (CH, C-2), 69.1 (CH, C-4'), 59.7 (CH$_2$, O$\underline{C}$H$_2$CH$_3$), 55.4 (CH), 52.7 (CH), 42.2 (C, C-7a'), 40.3 (CH$_2$), 39.2 (CH), 34.2 (CH$_2$), 27.2 (CH$_2$), 25.6 (3xCH$_3$, Me$_3$CSi), 22.8 (CH$_2$), 18.9 (CH$_3$, OCH$_2$$\underline{C}H_3$), 17.8 (C, Me$_3$CSi), 17.4 (CH$_2$), 14.1 (CH$_3$, C-5), 13.7 (CH$_3$, C-8'), -5.0 (CH$_3$, MeSi), -5.4 (CH$_3$, MeSi).

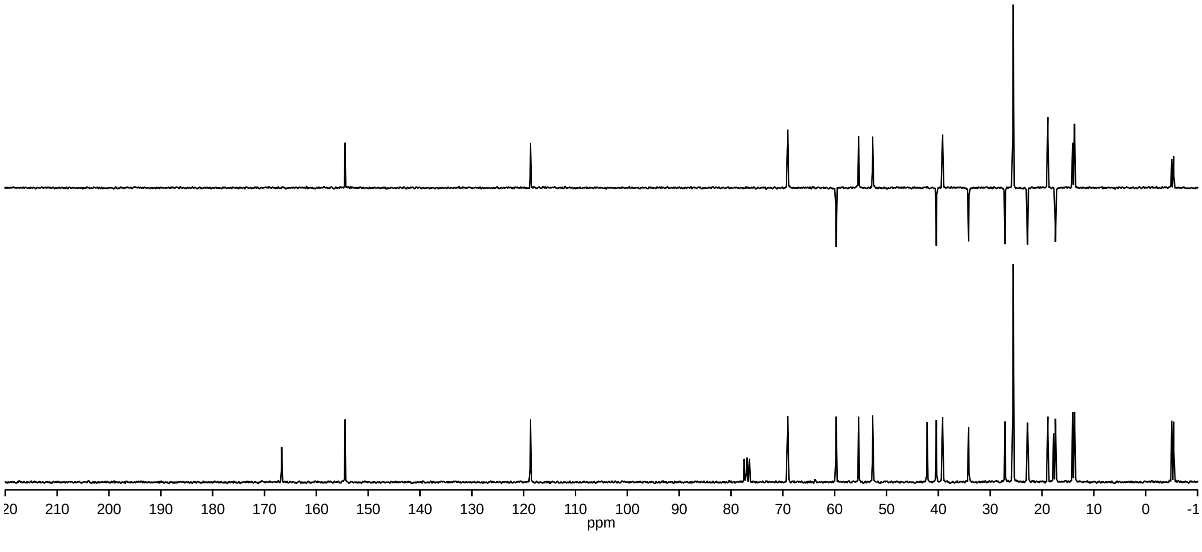

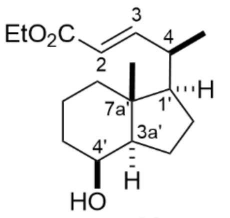

20

^{1}H NMR (250 MHz, $CDCl_3$): δ 6.78 (1H, dd, *J* = 15.6 Hz, *J* = 9.0 Hz, H-3), 5.70 (1H, d, *J* = 15.4 Hz, H-2), 4.13 (2H, c, *J* = 7.1 Hz, OC<u>H</u>$_2$CH$_3$), 4.04 (1H, s, H-4'), 1.24 (3H, t, *J* = 7.2 Hz, OCH$_2$C<u>H</u>$_3$), 1.03 (3H, d, *J* = 6.6 Hz, H-5), 0.93 (3H, s, CH$_3$-8').

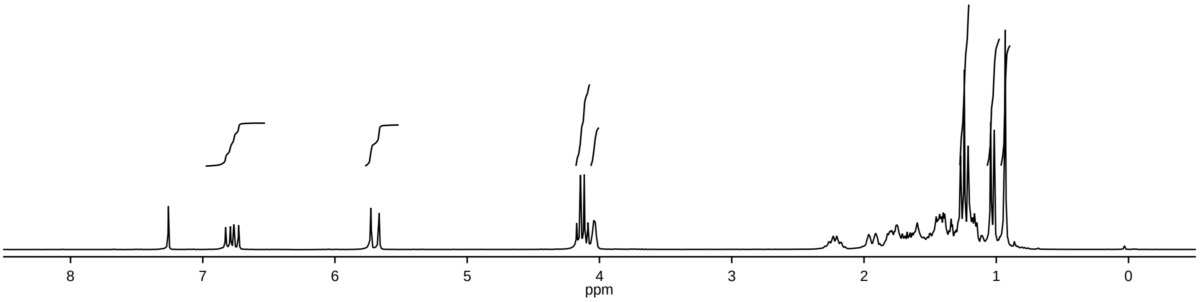

^{13}C NMR (63 MHz, $CDCl_3$): δ 166.9 (C, C=O), 154.5 (C, C-3), 118.8 (CH, C-2), 68.9 (CH, C-4'), 59.9 (CH$_2$), 52.2 (CH), 52.3 (CH), 41.9 (C, C-7a'), 40.1 (CH$_2$), 39.2 (CH), 33.4 (CH$_2$), 27.1 (CH$_2$), 22.3 (CH$_2$), 18.9 (CH$_3$), 17.8 (C, Me$_3$CSi), 17.3 (CH$_2$), 14.1 (CH$_3$, C-5), 13.6 (CH$_3$, C-8').

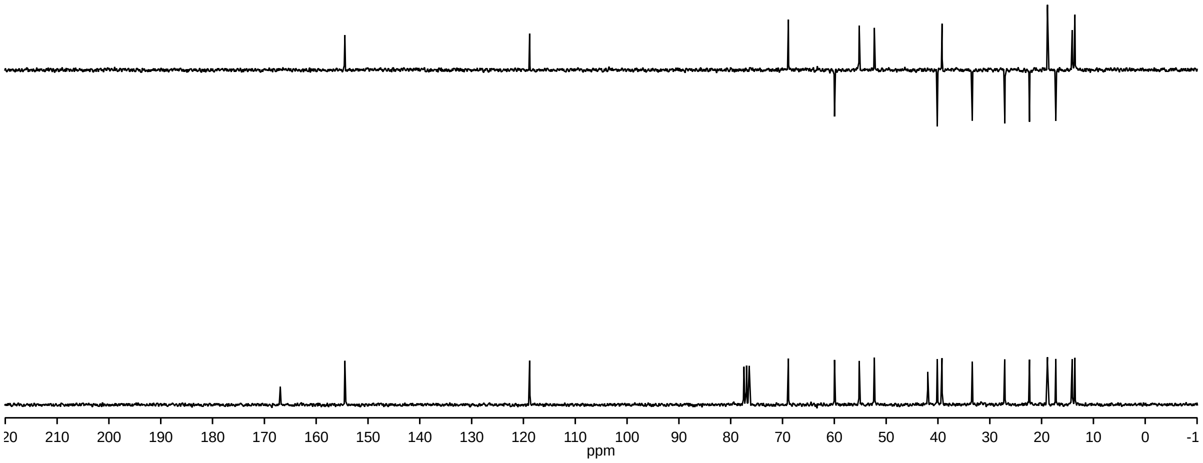

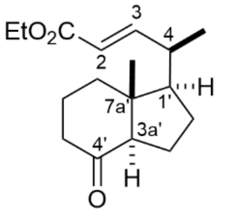

12

^{1}H NMR (250 MHz, $CDCl_3$): δ 6.79 (1H, dd, *J* = 15.6, *J* = 9.0, H-3), 5.72 (1H, d, *J* = 15.6 Hz, H-2), 4.14 (2H, c, *J* = 7.1 Hz, $OC\underline{H}_2CH_3$), 1.25 (3H, t, *J* = 7.2 Hz, $OCH_2C\underline{H}_3$), 1.09 (3H, d, *J* = 6.6 Hz, H-5), 0.64 (3H, s, CH_3-8').

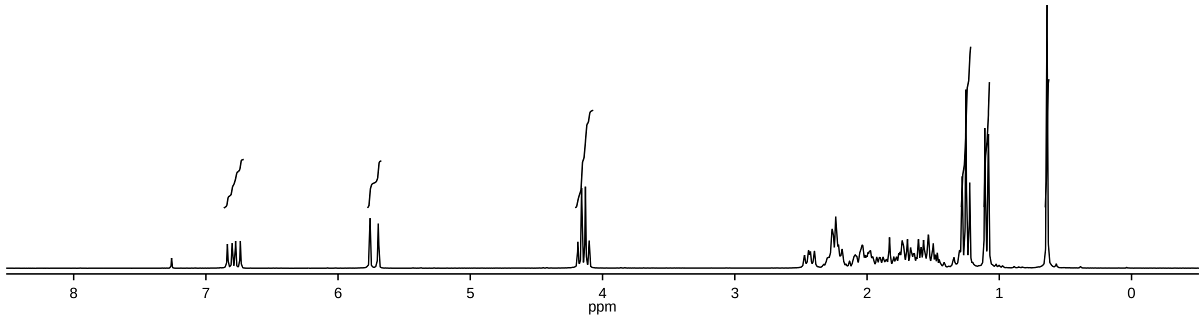

^{13}C NMR (63 MHz, $CDCl_3$): δ 211.2 (C, C=O), 166.8 (C, C=O), 153.3 (CH, C-3), 119.4 (CH, C-2), 61.5 (C, CH), 60.0 (CH_2), 55.3 (CH), 49.7 (C, C-7a'), 40.7 (CH_2), 39.2 (CH), 38.6 (CH_2), 27.1 (CH_2), 23.8 (CH_2), 19.2 (CH_3), 18.9 (CH_2), 14.1 (CH_3, C-5), 12.6 (CH_3, C-8').

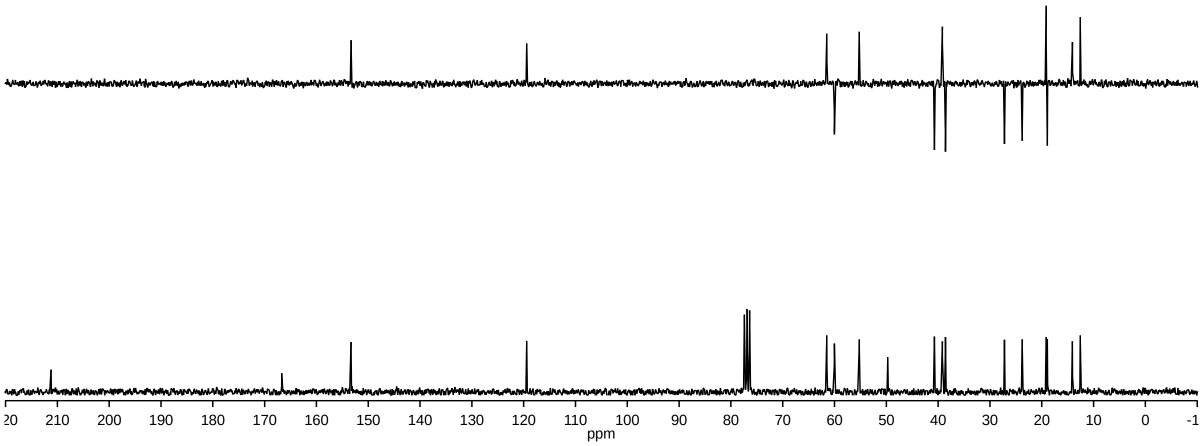

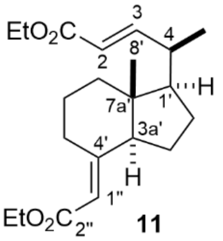

11

^{1}H NMR (250 MHz, $CDCl_3$): δ 6.74 (1H, dd, *J* = 15.6 Hz, *J* = 9.0 Hz, H-3), 5.72 (1H, d, *J* = 15.5 Hz, H-2), 5.42 (1H, d, *J* = 1.9 Hz, H-1''), 4.14 (4H, c, *J* = 8.5 Hz, 2x(OC<u>H</u>$_2$CH$_3$), 3.83 (1H, m), 2.42 (1H, m), 2.08 (1H, t, *J* = 9.4 Hz), 1.25 (6H, t, *J* = 8.5 Hz, 2x(OCH$_2$C<u>H</u>$_3$), 1.07 (3H, dd, *J* = 6.5 Hz, *J* = 1.8, H-5), 0.57 (3H, s, CH$_3$-8').

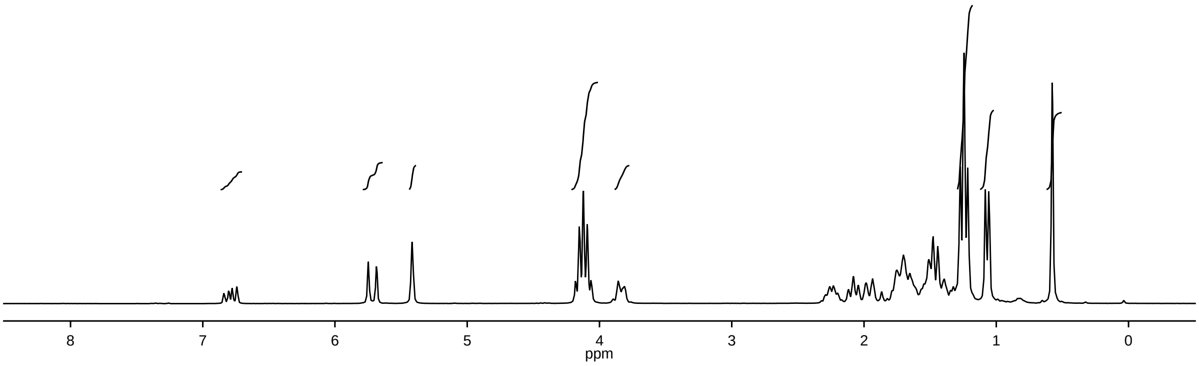

^{13}C NMR (63 MHz, $CDCl_3$): δ 166.7 (C, C=O), 166.6 (C, C=O), 162.4 (C, C-4'), 153.8 (C, C-3), 119.2 (CH, C-2), 112.1 (CH, C-1''), 59.9 (CH$_2$), 59.3 (CH$_2$), 56.4 (CH), 52.3 (CH), 46.9 (C, C-7a'), 39.7 (CH$_2$), 39.6 (CH), 29.3 (CH$_2$), 27.1 (CH$_2$), 23.5 (CH$_2$), 21.9 (CH$_2$), 19.2 (CH$_3$), 18.9 (CH$_2$), 14.1 (CH$_3$, C-5), 12.2 (CH$_3$, C-8').

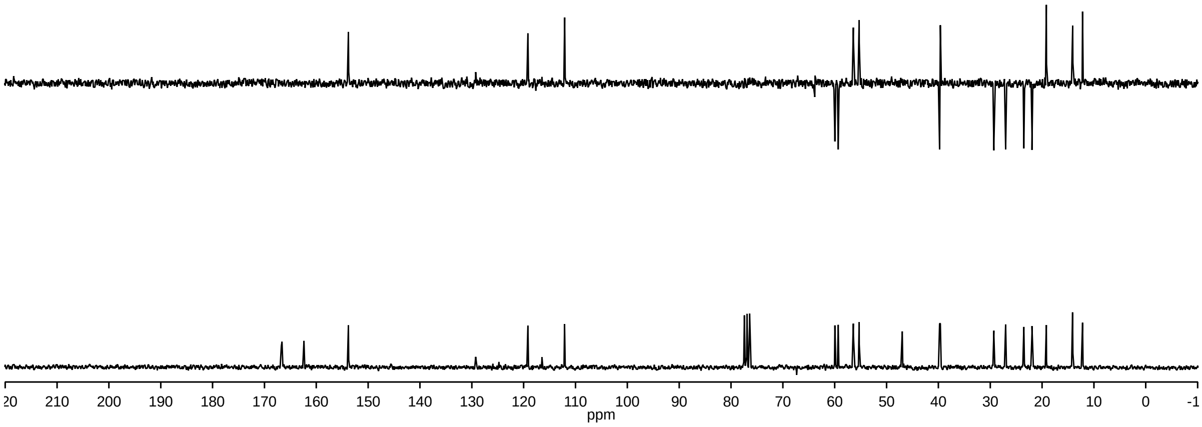

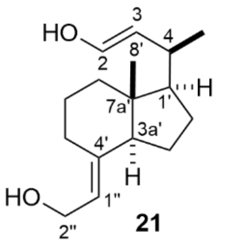

21

^{1}H NMR (250 MHz, $CDCl_3$): δ 5.49 (2H, m, H-2, H-3), 5.15 (1H, t, J = 6.9 Hz, H-1''), 4.14 (2H, d, J = 6.9 Hz), 4.00 (2H, d, J = 3.0 Hz, 2.57 (1H, m,), 1.00 (3H, d, J = 6.6 Hz, CH_3-5), 0.53 (3H, s, CH_3-8').

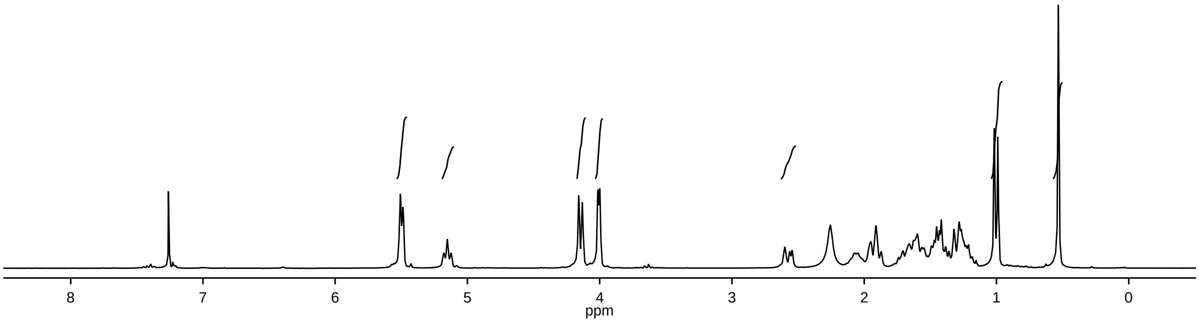

^{13}C NMR (63 MHz, $CDCl_3$): δ 142.8 (C, C-4'), 138.8 (CH, C-3), 126.5 (CH, C-2), 119.3 (CH, C-1''), 63.5 (CH_2), 58.3 (CH_2), 55.8 (CH), 55.4 (CH), 45.1 (C, C-7a'), 40.1 (CH_2), 39.7 (CH), 28.5 (CH_2), 27.5 (CH_2), 23.2 (CH_2), 21.9 (CH_2), 20.2 (CH_3, C-5), 18.9 (CH_3, C-8').

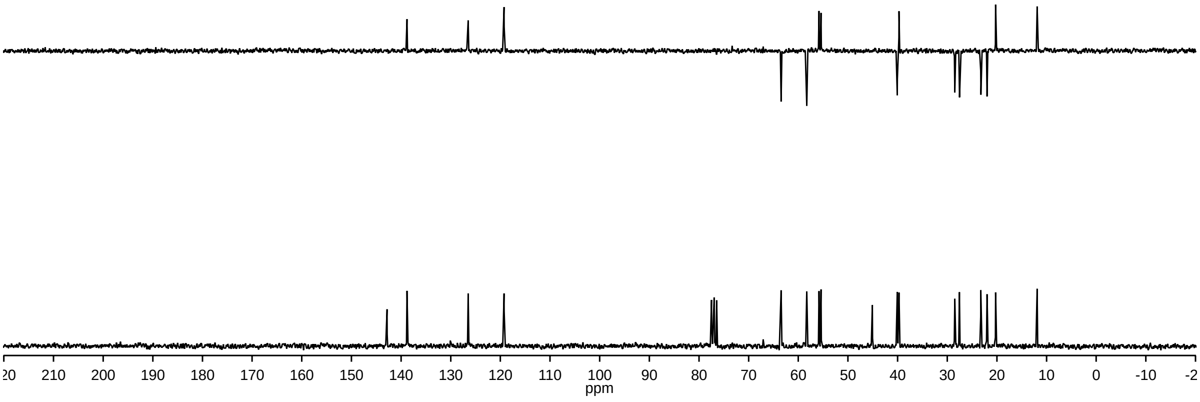

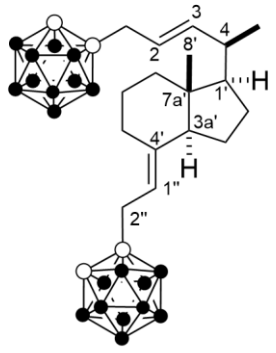

5

^{1}H NMR (500 MHz, $CDCl_3$): δ 5.39 (1H, dd, *J* = 15.1 Hz, *J* = 8.6 Hz), 5.24 (1H, dt, *J* = 15.0 Hz, *J* = 7.3 Hz), 4.86 (1H, t, *J* = 8.1 Hz), 3.54 (2H, s, HC-Carb), 2.99 (3H, qd, *J* = 15.0 Hz, *J* = 8.1 Hz), 2.86 (2H, d, *J* = 7.3 Hz), 2.45 (2H, d, *J* = 11.3 Hz), 1.03 (3H, s, CH_3-5), 0.55 (3H, s, CH_3-8').

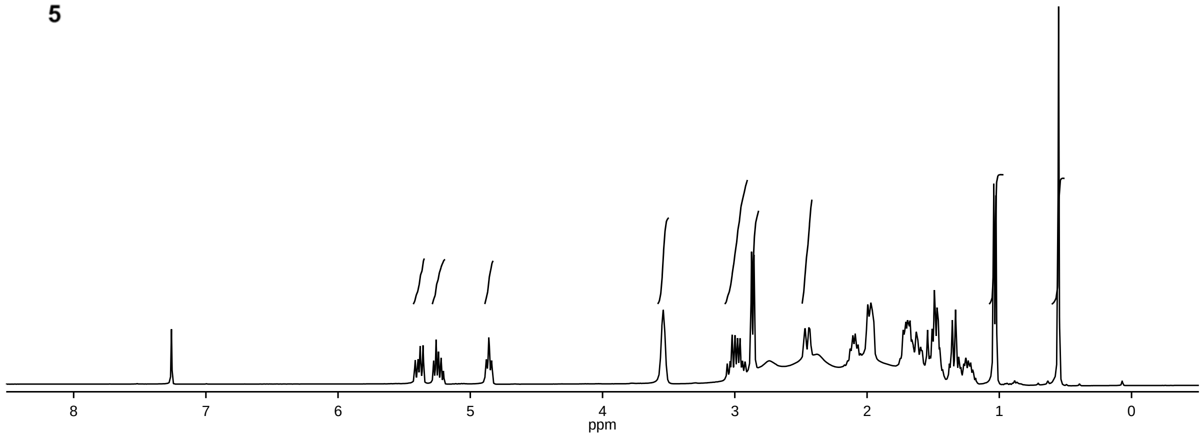

^{13}C NMR (63 MHz, $CDCl_3$): δ 145.1 (C, C-4'), 143.2 (CH), 120.5 (CH), 113.2 (CH), 79.9 (C), 74.4 (C), 59.3 (CH), 55.5 (CH), 55.4 (CH), 45.3 (C, C-7a'), 40.6 (CH_2), 40.1 (CH), 39.8 (CH_2), 34.7 (CH_2), 28.4 (CH_2), 27.6 (CH_2), 23.2 (CH_2), 22.1 (CH_2), 20.3 (CH_3, C-5), 12.0 (CH_3, C-8').

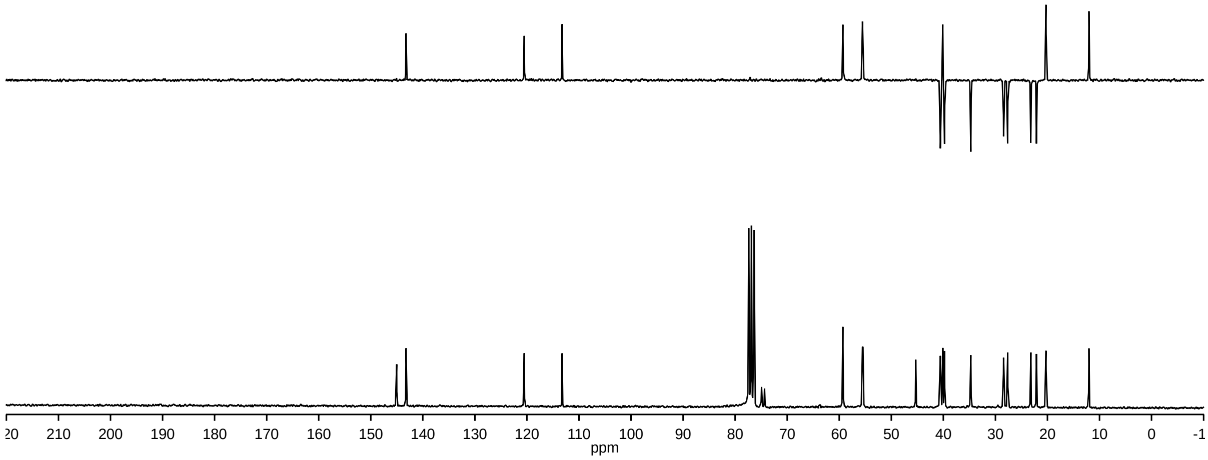

^{11}B NMRc (160.46 MHz, $CDCl_3$): δ -2.96, -3.88, -6.32, -7.27, -9.71, -10.63, -11-51, -12.54, -13.69, -14.21.

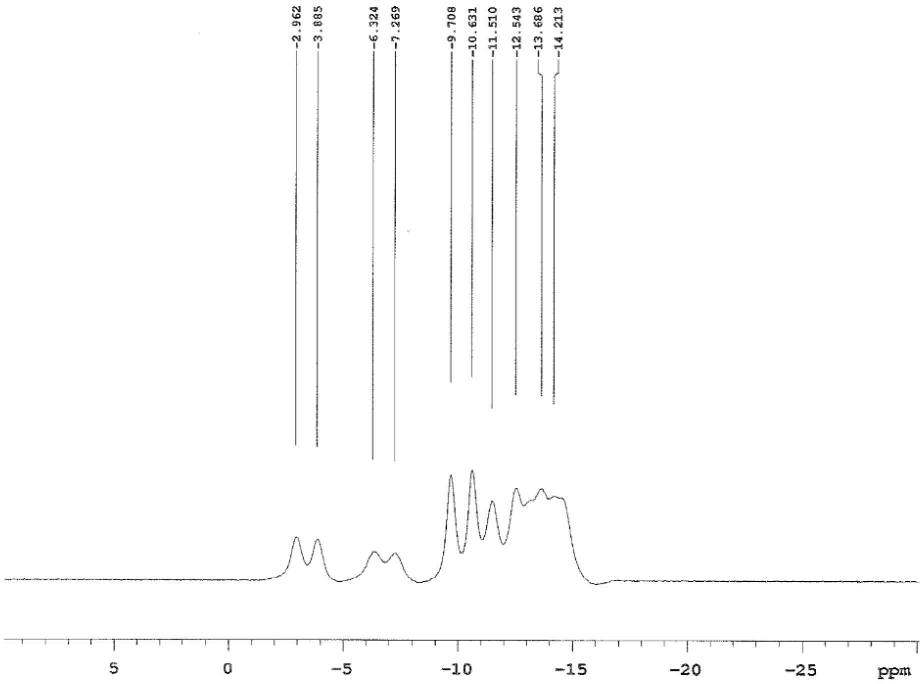

^{11}B NMRdec (160.46 MHz, $CDCl_3$): δ -3.42, -6.81, -10.17, -12.01, -14.08.

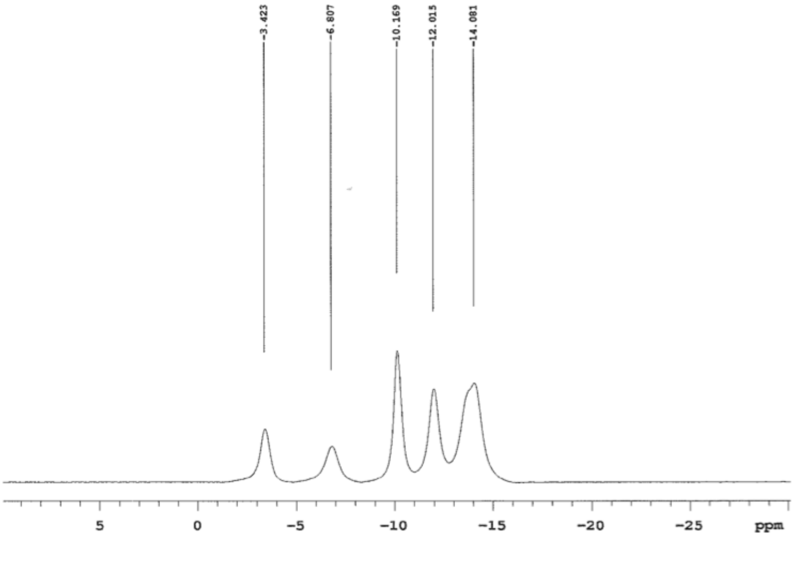

Figure SI-1. Superimposition Compound 4 and 1,25D$_3$

The corresponding superimposition of analogue **4** (magenta) with 1,25D$_3$ (**1**) (yellow).

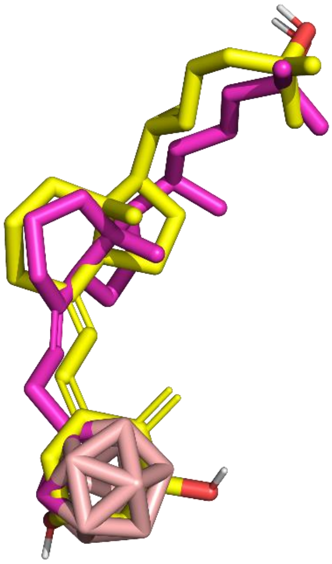

Table SI-1. Serum calcium levels in mice, mRNA expression of the gene encoding CYP24A1, and cytotoxicity (MTT assay) of 1,25D$_3$, compound **4** and compound **5**. Mice were treated intraperitoneally with sesame oil (control) and with 1,25D$_3$, compound **4** and compound **5** at doses of 0.3 micrograms/kg body weight every other day for 21 days. Blood was drawn to assess calcaemia and total RNA was obtained from tail tissue of mice to assess CYP24A1 mRNA expression by real-time PCR. An MTT assay was performed to assess cell cytotoxicity in MCF-7 human breast adenocarcinoma cells after 48 h of treatment.

			**MTT metabolization (A_{570})**		
	**Serum Ca^{++} levels (mg/dl)**	**CYP24A1 mRNA expression (RU)**	[1 nM]	[10 nM]	[100 nM]
**Control**	7.07 ± 0.67	3.94 ± 2.09	0.41 ± 0.003	0.41 ± 0.003	0.41 ± 0.003
**1,25D$_3$**	8.34 ± 0.27	31.27 ± 8.49	0.41 ± 0.005	0.38 ± 0.010	0.33 ± 0.004
**compound 4**	7.93 ± 0.23	21.89 ± 5.45	0.38 ± 0.005	0.37 ± 0.007	0.32 ± 0.011
**compound 5**	8.16 ± 0.23	6.42 ± 6.47	0.39 ± 0.008	0.35 ± 0.007	0.33 ± 0.006
